# Letter to the Editor on ‘Smoking cessation in pregnancy: An update for maternity care practitioners’

**DOI:** 10.18332/tid/116834

**Published:** 2020-03-26

**Authors:** Alain Braillon

**Affiliations:** 1 University Hospital, Amiens, France

**Keywords:** ‘agnotology’, 5As, pharmacotherapy, nicotine substitute

**Dear Editor,**

The concern for improving smoking cessation in pregnant women^1^ is most welcome but deserves comment, despite rightly stressing the mandatory needs for: a) trained providers or specialized services; and b) the belt-and-braces strategy, combining psychological support with pharmacotherapy (nicotine substitutes using patches plus lozenges)^1^.

Indeed, the present state of affairs is a ‘shipwreck’: a) in the US, only 1 in 5 pregnant smokers is offered nicotine substitutes, and 1 in 4 does not receive any intervention to quit^2^; and b) In France, during the World No-Tobacco Day, in the main entrance of a university hospital, midwives ran a booth with the banner: ‘*Acupuncture, an alternative to nicotine replacement therapy in smoking cessation*’^3^. As a matter of fact, since 2017, nicotine substitutes packages have a big red label that reads *‘nicotine + pregnancy = danger’* and a pictogram with a black subtitle that reads *‘do not use during pregnancy unless no therapeutic alternative’*. Worse, the French Department of Health flied in the face of evidence and common sense, even rejected pledges by the Institute of Medicine and NGOs to withdraw these devastating warnings^4^. Moreover, warnings are ‘icing on the cake’ as too many professionals overlook: a) the deleterious effects of compensatory uptake of harmful by-products (CO, tar, etc.) when trying to quit without nicotine substitutes^5^; and b) that smoking with nicotine substitutes does not cause an increase in nicotine concentration, and they overestimate by a factor of 10 the nicotine deadly dose^6^. Frequently, at follow-up visits my patients report that their pharmacists warned them against smoking with patches or challenged my prescriptions as being too high; with my warnings to pharmacist and medical regional councils usually being ignored.

However, the International Health Maintenance Organizations’ 5 As (Ask, Advise, Assess, Assist, Arrange) approach is flawed, it is the opposite of the motivational interviewing that is rightly promoted by Diamanti et al.^1^. No need to Ask, look at fingers and smell. Advising is pointing the finger of blame, as though these women are dumb; advising to quit can only decrease further an already poor self-esteem. Why Assessing readiness to quit? Tobacco is the worst drug and no smoker expects to be able to quit. All smokers fear quitting, having made a series of attempts, with always the same results: suffering and despair.

First, one needs to simply reassure and reassure: ‘I do not require you to quit, but only to take the treatment of smoking with patches, which is less dangerous than without, and do increase the dose as needed’; craving and desire to smoke result in pain and suffering due to lack of nicotine; ‘Hasten slowly, you will naturally quit when there is no craving and cigarettes become distasteful’. Indeed, fixing a quit-date is a programmed failure! Can doctors set a date for their patients to become pain free? Second, one needs to educate smokers to self-increase the dose of nicotine substitutes (patches and faster-acting forms) until the craving is suppressed and cigarettes become distasteful. Third, there is no evidence yet for the efficacy of e-cigarettes versus proactive evidence-based treatment. Switching to vapor does not qualify as cessation^7^; and e-cigarette smoke in mice produces DNA damage, inhibits DNA repair, causes lung adenocarcinomas, and bladder urothelial hyperplasia^8^.

Proctor^9^ created the term ‘agnotology’ (the study of culturally induced ignorance or doubt) to highlight the misrepresentation of facts by the tobacco industry to fool ordinary people. This seems to be a case of the ‘pot calling the kettle black’.
